# Enhancing public legal awareness in regulating normative deviance: a social psychological evaluation of legal education

**DOI:** 10.3389/fpsyg.2026.1856345

**Published:** 2026-09-17

**Authors:** He Liu, Jun Li

**Affiliations:** 1 School of Law, Xinjiang University, Ürümqi, Xinjiang, China; 2 School of Law, Yili Normal University, Yining, Xinjiang, China

**Keywords:** before-after evaluation, deviant behavior, legal education, public legal awareness, social psychology, theory of planned behavior

## Abstract

Public legal awareness is a key factor in regulating deviant behavior, and legal education is the main channel for improving public legal awareness. This study evaluates the importance of raising public legal awareness in regulating deviant behavior from the perspective of social psychology, using legal socialization theory and planned behavior theory as conceptual frameworks. This paper first adopts a pre - and post evaluation design in six cities in a province, and then uses effective scales and public perception of legal functions to measure the four core structures of the Theory of Planned Behavior - attitude towards legal compliance, subjective norms, perceived behavioral control, and behavioral intention. The results showed that the total number of abnormal events in the six cities decreased from 274 to 102, and the paired differences were statistically significant, *t*(5)=9.40, *p*<0.001; the age distribution of the participating group had undergone significant changes, with chi square (3)=15.62 and *p*=0.001; the distribution of risk levels had undergone significant changes, with chi square (2)=150.48, *p*<0.001. Research has shown that at the individual level, attitudes, subjective norms, perceived behavioral control, and behavioral intentions all show significant improvement after the project (all *p*<0.05), and these psychological structures significantly mediate the association between legal education and reducing deviant behavioral tendencies (indirect effect=–0.643, 95% CI [–0.736, –0.556]). Although the design does not allow for causal attribution, legal education is associated with a significant reduction in deviant behavior and a positive shift in public legal awareness through identifiable psychological mechanisms.

## 1 Introduction

Due to the low level of economic development at this stage, citizens’ awareness of the legal system is relatively weak. This makes people’s legal awareness relatively weak, and this legal quality is often affected by some negative concepts in traditional culture, resulting in people’s lack of due legal concepts and normative behaviors. Many people who do not know the truth tend to blindly follow the crowd and make deviant behaviors, which have a bad impact on society. Based on this, it is extremely important to analyze the public’s legal consciousness and deviant behavior and explore its psychological significance for social development and the construction of a legal society.

Deviant behaviors often appear in society, which arouses the attention of social groups and sociology experts. Yam et al. (2017) derived the potential negative consequences of Organizational Citizenship Behavior (OCB) from self-determination and moral licensing theories, proving that OCB generates a sense of entitlement that transcends organizational boundaries and leads to biases outside the organization. Kruglanski et al. (2017) outlined a general psychological theory of extremism and applied it to the special case of Violent Extremism (VE). Turel (2017) aimed to examine the antecedents of the negative impact of social networking sites on individuals and organizations as a means to point to future interventions. Ji et al. (2019) analyzed 741 small and medium-sized business owners in China. The results showed that materialism and trust in institutional justice directly or indirectly affect business bias. Cammaerts and Decillia (2020) research found everything from traditional surveillance practices to ranting attacks is unhealthy for democracy, and it also raises serious ethical questions about UK journalism and its role in society. Krylova et al. (2017) reviewed the state of knowledge about integrity-based offenses and highlighted the leader-follower relationship. Research by Shuman et al. (2018) has shown that image threats can be an effective motivating factor for high-identification people to address in-group delinquency. The frequent occurrence of illegal and criminal incidents in the society has caused social panic and caused psychological pressure to the masses.

Social psychology has become a hot topic in recent years, and many scholars have studied social psychology. Fang et al. (2019) discussed the theoretical and practical implications of understanding the psychological mechanisms of prosocial behavior. Pitts (2019) proposed that communication taste is a new dimension of social taste, which has theoretical and practical significance in the fields of positive communication, language and social psychology. Humberg et al. (2018) described the mathematical basis required to interpret the results of reaction surface analysis and provided a checklist for correctly identifying congruential effects. Kelty-Stephen (2017) examined preschool children’s mastery of the cooperative principle adage and the social cognitive scaffolding that supports this mastery. The results showed that children consistently identify violations of the principle of cooperation (Kelty-Stephen, 2017). Banerjee et al. (2021) explored the relevance of attribution in the field of psychosocial health and described the understanding and application of related theories in stigma research and consumerism. Brannigan (2021) discussed the archival reassessment of the researcher’s work. Liem and Hall (2020) introduced the potential use of respondent-driven sampling methods in social psychology research. Social psychology is often used to analyze social phenomena, yet few studies have applied its core constructs to evaluate whether public legal awareness is strengthened through legal education and whether such strengthening is associated with reduced deviant behavior at the community level (Ajzen, 1991; Bandura, 1977). Legal socialization theory holds that repeated exposure to legal rules and institutions promotes the internalization of the legitimacy of legal authorities, and that this internalization shapes law-related attitudes and subsequent behavior (Fagan and Tyler, 2005; Tyler, 2003). The theory of planned behavior specifies that attitudes toward a behavior shape behavioral intention and, in turn, behavior, providing a framework for understanding how legal education influences legal awareness and deviant conduct through attitude change. Within this framework, legal education operates as a socialization channel that transmits legal knowledge and shapes attitudes toward legal norms, and deviant behavior is regulated through the internalization of legal norms rather than through deterrence alone. This theoretical account grounds the relationship between legal awareness and deviant behavior in established social psychological constructs and provides the analytical basis for the present study. Drawing on the theory of planned behavior, the present study operationalizes the psychological mechanism of legal education through four core constructs: (a) attitude toward legal compliance, defined as the individual’s favorable or unfavorable evaluation of complying with legal norms; (b) subjective norm, defined as the perceived social expectation and pressure from significant others and the community to comply with the law; (c) perceived behavioral control, defined as the perceived ease or difficulty of complying with the law and fulfilling obligations; and (d) behavioral intention, defined as the intention to comply with the law, fulfill obligations, and learn legal knowledge in the near future. Accordingly, we hypothesize that (H1) legal education is associated with significant improvements in attitude toward legal compliance, subjective norm, perceived behavioral control, and behavioral intention; (H2) attitude, subjective norm, and perceived behavioral control positively predict behavioral intention; and (H3) the TPB constructs mediate the association between legal education and reduced deviant behavior tendency.

In order to improve residents’ legal awareness, the present study evaluated the importance of enhancing public legal awareness in regulating deviant behavior from the perspective of social psychology. The research questions concerned whether the number of deviant incidents in the surveyed cities decreased after the implementation of legal education, whether the age distribution of the groups involved in deviant incidents changed, and whether public perceptions of the functions of law shifted toward the protection of rights and the performance of obligations. The study drew conclusions from a quantitative comparison of deviance incident records and from a survey of public perceptions conducted before and after the implementation of legal education, and the findings provide a theoretical and practical basis for improving public legal awareness and regulating deviant behavior.

## 2 Relevant evaluation on legal awareness

Legal awareness is a general term for people’s views, knowledge and psychological attitudes toward the law and related legal phenomena (Michela, 2021). Legal awareness is a conceptual legal culture that is very important to the formation and application of law. It is manifested in various legal theories that study legal phenomena, in the evaluation and interpretation of existing laws, in people’s legal motives, in their awareness of rights and obligations, in their understanding, mastery and application of laws and legal systems, and in their evaluation of the legitimacy of their actions.

### 2.1 Public legal awareness

Public legal awareness refers to the public’s attitudes and views on legal issues (Makwae, 2021). It has a certain homogeneity and consistency in legal awareness, depends on the development of the public and public opinion, represents the general public’s views on various legal issues, and is often reflected in the public’s thinking and attitudes toward various legal issues. The public’s legal awareness is mainly expressed through the perceptual feelings and cognition of the law, but also has a rational view of the law.

### 2.2 Characteristics of public legal awareness

The public legal consciousness is different from the general legal consciousness and has particularity. Its characteristics can be seen in Figure 1.

**Figure 1:**
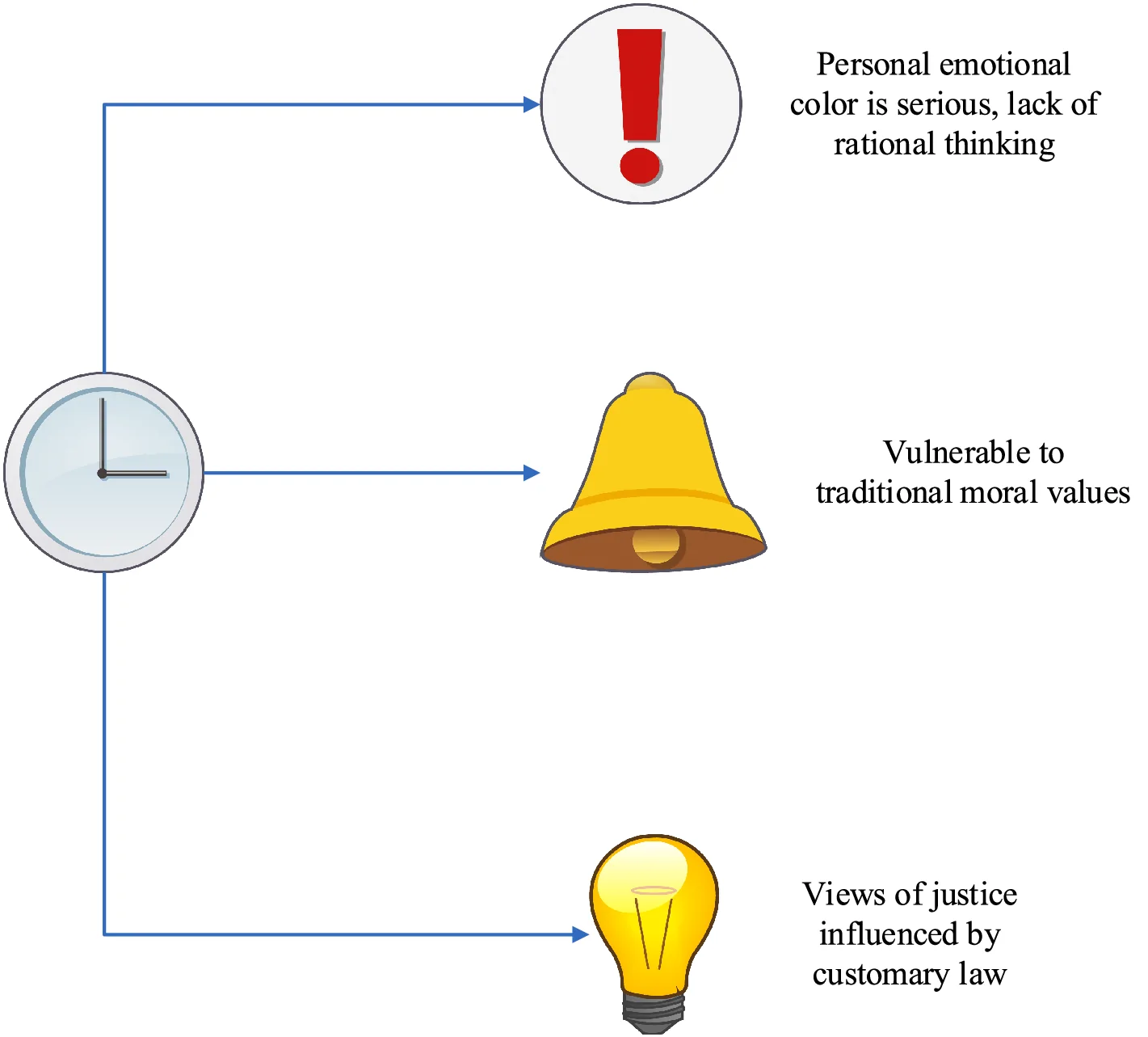
Characteristics of public legal awareness.

#### 2.2.1 Severe personal emotions and lack of rational thinking

The public’s legal awareness often has a personal emotional color, and people’s evaluation of hot events is often irrational and not based on legal provisions. This phenomenon is relatively common on the Internet (Paul, 2021). Some unscrupulous people on the Internet freely criticize hot events and interpret issues one-sidedly. Many people who do not know the truth imitate what others say, and criticize and speak, which has caused a relatively bad social impact. When people discuss topics, they do not look at problems with rational thinking and pursue the truth based on the law. Instead, they follow the majority of irrational people to think irrationally and make irrational behaviors. Such phenomena have aroused widespread concern among social groups.

#### 2.2.2 Easily influenced by traditional moral concepts

The legal thought of the three cardinal guides and five constant virtues inherited from ancient times affects the implementation and development of contemporary legal provisions (Singh, 2020). In today’s society, many people still follow the old, outdated, and corrupt ideas that hinder the progress of the current society. They feel that violating morality and public order and good customs is a violation of the law, so they make irrational behaviors and cause bad consequences. Because of the lack of legal knowledge that meets the current requirements, the public would be easily influenced by traditional moral concepts and make wrong judgments on legal events.

#### 2.2.3 The concept of justice is influenced by customary law

The public’s view of justice is often influenced by customary law. Customary law is a set of behavioral norms that does not depend on national laws. It is based on certain social authority and social organization and has certain coercive force. It is neither a pure moral norm nor a complete legal norm, but a quasi-legal norm between morality and law. Customary law functions as a de facto legal norm in the public consciousness. Retaliatory practices such as taking a life for a life or enforcing debt repayment through private means are recognized under customary law yet are not sanctioned by statutory law. Statutory law imposes distinct requirements across different categories of cases and requires adjudication according to codified legal provisions.

## 3 Concrete evaluation of deviant behavior

Fault occurs when a person harms himself, another person, the environment, or a living being, and when he or she fails to take action when he or she encounters something that is harmful (Sarita, 2020). Deviance is a social act and disobedience to a set of norms established and accepted by the majority of people in a community or society. In a given economic and cultural context, any thought or behavior that is considered by community members to violate their social norms or values is a deviance. Deviance includes all thoughts and actions that are criminal, illegal and contrary to moral standards, customs and social norms. It is understood in terms of the society’s explanatory system in which it occurs.

### 3.1 Characteristics of deviant behavior

Deviance has the following characteristics, which can be seen in Figure 2:

1) It is relative;
2) It must violate important norms;
3) It is a behavior that most people disapprove of;
4) It is not exactly equivalent to a social problem;
5) The degree of deviance and the degree of punishment depends on the seriousness of the violation of the norm.

**Figure 2:**
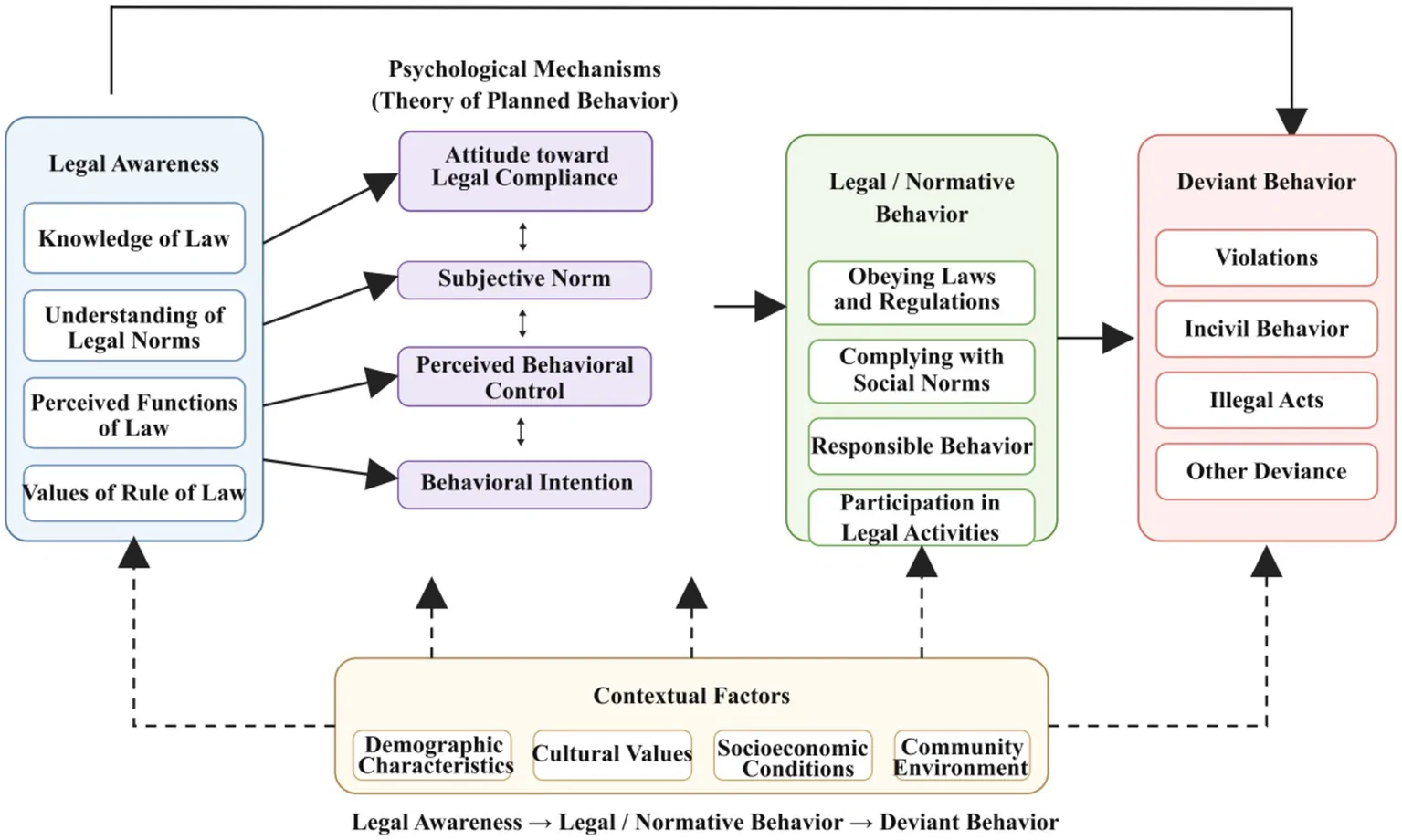
Characteristics of deviance.

### 3.2 Classification of deviant behavior

Deviance can be divided into five categories according to the severity: inappropriate behavior, abnormal behavior, unethical behavior, anti-social behavior, and criminal behavior, as shown in Figure 3.

**Figure 3:**
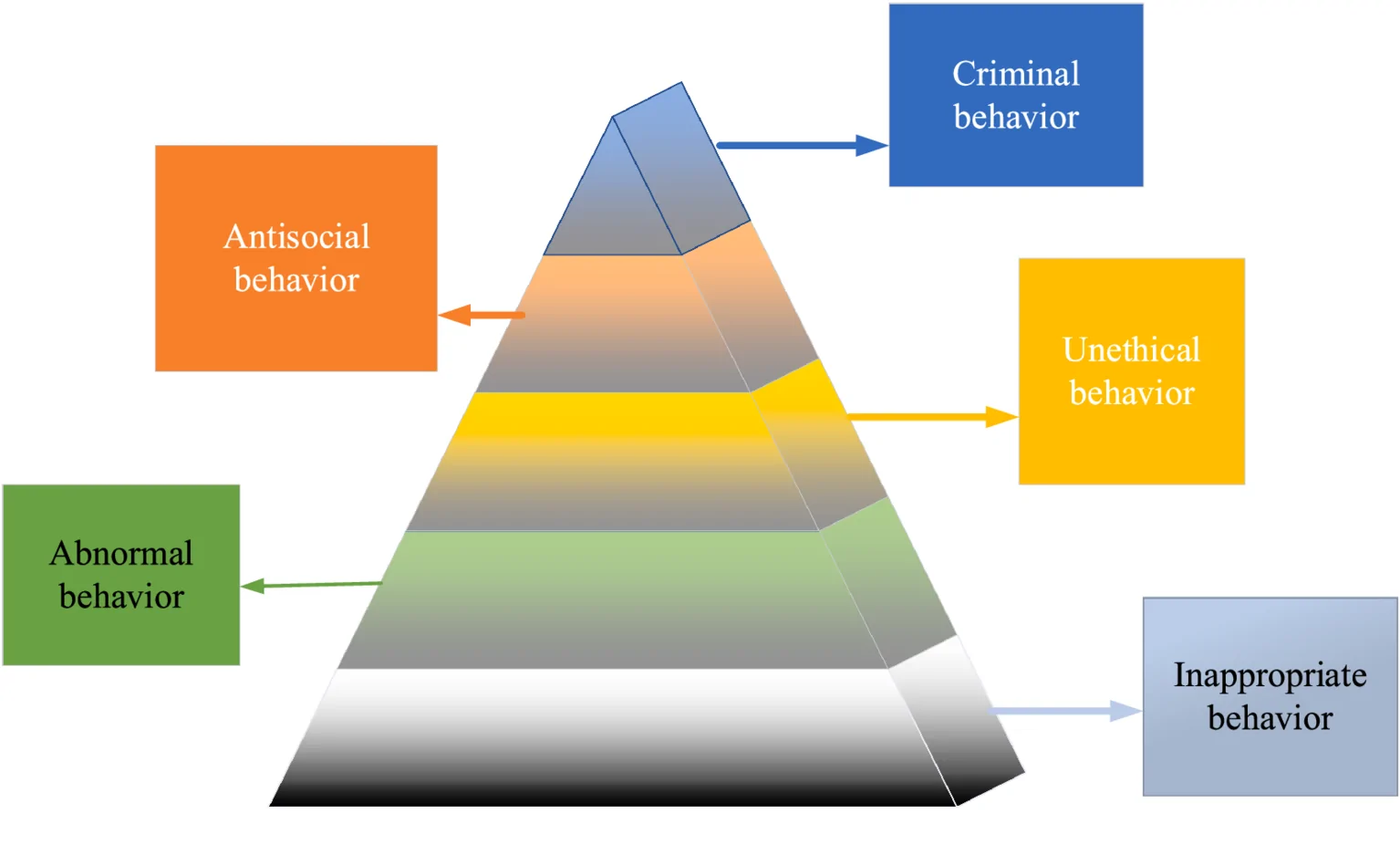
Classification of deviant behavior.

Inappropriate conduct is conduct that violates the rules applicable to a particular case without causing significant harm to society. This is an act that causes resentment, but is usually not formally punished; abnormal behavior: this is behavior that is usually a violation of social norms and is caused by mental illness or psychopathology; immoral behavior: this is behavior that violates the generally accepted norms of life and behavior, and is generally condemned by public opinion; anti-social behavior is an act that causes harm, even serious harm, to others and society; criminal behavior is antisocial behavior that is punished as a criminal offense. The classification follows the normative tradition in the sociology of deviance, in which deviant acts are ordered along a continuum of social reaction from the violation of conventional expectations to the violation of codified criminal norms (Heckert and Heckert, 2002).

Inappropriate conduct reflects the sociological conception of a violation of conventional expectations; abnormal behavior represents the psychological and psychiatric conception of norm violation rooted in mental disorder; immoral behavior characterizes the ethical conception of conduct condemned by shared moral standards; antisocial behavior embodies the criminological conception of harm directed toward others and society; criminal behavior denotes the legal conception of conduct codified as a punishable offense.

### 3.3 The danger of deviant behavior

The harm of deviant behavior is as follows: causing alienation and disharmony between members of society and between groups and individuals; destroying social order and affecting social stability (Andrews, 2020). It has a great impact on people’s physical and mental development, and seriously hinders the normal social and economic activities and the stability and security of social order; it causes chaos in family life, which is not conducive to the healthy growth of the next generation; it destroys the social atmosphere and hinders the progress of socialist modernization.

#### 3.3.1 Disrupting the social order

Deviance events occur frequently in social life and affect not only the individuals directly involved but also other members of the social group. Unresolved grievances and perceived injustices foster collective resistance within social groups, producing adverse effects on social development, disrupting the operation of organizations, and damaging social order.

#### 3.3.2 Disrupting the normal life order

The occurrence of a deviant incident influences public perception and behavior. Unfair handling of social events attracts public attention and discussion, disrupting the normal order of life. When rule violations go unpunished, observers may perceive that compliance is not enforced, weakening the normative constraint on behavior and increasing the likelihood of further violations. The erosion of legal norms contributes to additional deviant acts and destabilizes public life.

#### 3.3.3 Damaging the legitimate rights and interests of others

The occurrence of deviant behaviors is often accompanied by damage to the legitimate rights and interests of others, bringing property or life threats to others. If everyone does not abide by traffic laws and acts illegally, the safety of people’s lives would not be guaranteed. Only when everyone abides by the traffic laws and regulates their own behavior, can the safety of everyone’s property and life be guaranteed.

## 4 Data and statistical methods

### 4.1 Study design and setting

A before-after evaluation design without a control group was employed in this study. The study was conducted in a province of China, a civil law jurisdiction in which public legal education is institutionalized through the national legal publicity and education campaign administered by judicial administration departments at each level of government. Under this campaign, community legal education programs are delivered regularly in cities and counties, and the six surveyed cities implemented such a program during the study period. The demographic and economic conditions of the province are comparable to those of other provinces in the eastern and central regions of China, and the findings are informative for other jurisdictions that operate community-based legal education programs within a civil law tradition.

### 4.2 Sample and sampling procedure

Six prefecture-level cities were drawn from the province by simple random sampling from the official administrative list of cities. All deviant incidents recorded by the public security organs of the six cities within the two investigation quarters were included in the incident statistics, and the inclusion criteria for incident records followed the formal case-filing categories of the public security statistics system. The survey respondents were permanent residents of the six cities aged 18 years and above.

### 4.3 The legal education program

The legal education program was implemented over a continuous period of 12 months in all six cities under the coordination of the local judicial administration departments. The evaluation covered the quarter immediately preceding the start of the program and the quarter immediately following its completion. The program delivered a standardized curriculum of community lectures and seminars covering constitutional law, civil law, criminal law, and the rights and obligations of citizens, together with case-based discussion sessions on typical deviant incidents. The instructors were legal professionals from the judicial administration departments and law faculties of local universities. The pedagogical strategy combined classroom lectures, the distribution of legal education materials, and community outreach activities, and the program was delivered to residents in the communities, schools, and workplaces of each city.

### 4.4 Survey instrument and measurement

A structured questionnaire was developed to measure the four core constructs of the theory of planned behavior (TPB) and the perceived effect of legal education. The TPB items were adapted from the standard TPB questionnaire construction guidelines (Ajzen, 2002; Francis et al., 2004) and were rated on 7-point scales. Attitude toward legal compliance was measured with four semantic-differential items (e.g., “Complying with laws and regulations is: good–bad, beneficial–harmful, wise–foolish, pleasant–unpleasant”); subjective norm was measured with three items (e.g., “Most people who are important to me think I should comply with the law”); perceived behavioral control was measured with three items (e.g., “I am confident that I can comply with laws and regulations in daily life”); and behavioral intention was measured with three items (e.g., “In the next 3 months, I intend to strictly comply with laws and regulations”). Self-reported deviant behavior tendency was measured with three reverse-scored items (e.g., “I might violate social norms when no one is watching”). The questionnaire also retained the items on public perceptions of the functions of law and the perceived effect of legal education. The questionnaire was reviewed by two legal scholars and two social psychologists for content validity and was revised according to the comments of the review panel before administration. The questionnaire was administered face-to-face by trained investigators in the six cities in two waves, one conducted before the implementation of the legal education program and the other after it, and respondents were recruited by quota sampling according to the age and gender composition of the resident population of each city. A total of 1,000 valid questionnaires were collected, and the response rate reached 85.2%. Internal consistency was assessed with Cronbach’s alpha for each subscale (attitude toward legal compliance: *α* = 0.89; subjective norm: *α* = 0.85; perceived behavioral control: *α* = 0.82; behavioral intention: *α* = 0.86; deviant behavior tendency: *α* = 0.80). Confirmatory factor analysis supported the four-factor structure of the TPB measures, *χ*^2^(59) = 148.96, CFI = 0.95, TLI = 0.94, RMSEA = 0.049, indicating acceptable discriminant validity among the constructs.

### 4.5 Statistical analysis

Descriptive statistics were computed for the number of deviant incidents, the age distribution of the groups involved, the hazard grades of incidents, and the percentages of public perceptions. The comparison of incident numbers between the two quarters was tested with a paired-samples *t*-test, and the normality of the paired differences was checked with the Shapiro–Wilk test. The Wilcoxon signed-rank test was applied as a nonparametric complement, and the result was compared with that of the paired t test to assess the robustness of the parametric inference. The association between the implementation of legal education and the age distribution of the groups involved was tested with a chi-square test of independence, and the same test was applied to the distribution of hazard grades. Effect sizes were reported as Cohen’s d for the paired comparison and Cramér’s *V* for the chi-square tests, and the significance level was set at 0.05. To examine the psychological mechanism, the individual-level survey data from the two waves were pooled. Differences in the four TPB constructs and self-reported deviant behavior tendency between the pre- and post-program waves were tested with independent-samples *t*-tests, with effect sizes reported as Cohen’s *d*. Mediation analyses were conducted with the PROCESS macro (Model 4) using 5,000 bootstrap resamples, in which the pre/post-program wave was the independent variable, attitude toward legal compliance, subjective norm, and perceived behavioral control were parallel mediators, behavioral intention was the proximal mediator, and self-reported deviant behavior tendency was the outcome. Indirect effects were considered significant when the bias-corrected 95% bootstrap confidence interval excluded zero.

The incident statistics were compiled at three levels of aggregation. Table 1 reports the number of incident case files recorded by the public security organs in each city. Table 2 reports the number of individuals involved in these incidents, aggregated by age group; because a single incident case file may involve multiple individuals, the total in Table 2 exceeds the total in Table 1. Table 3 reports the number of separate deviant acts recorded within these case files, categorized by hazard grade; because a single case file may document multiple deviant acts, the total in Table 3 exceeds the totals in Tables 1, 2.

**Table 1:** Comparison of the number of deviant incidents before and after the implementation of legal education.

City	Before	After	Reduction	Reduction rate
City 1	36	12	24	66.70%
City 2	47	21	26	55.30%
City 3	39	15	24	61.50%
City 4	56	23	33	58.90%
City 5	68	26	42	61.80%
City 6	28	5	23	82.10%
Total	274	102	172	62.80%

Paired-samples *t*-test, *t*(5) = 9.40, *p* < 0.001; 95% confidence interval for the mean reduction, 20.82 to 36.51; Cohen’s *d*, 3.84; Wilcoxon signed-rank test, *p* = 0.031.

**Table 2:** Number of deviant incidents by group before and after the implementation of legal education.

Group	Before	After	Reduction rate
Adolescents	23	0	100.0%
Young people	68	26	61.8%
Middle-aged	157	84	46.5%
Older people	48	34	29.2%
Total	296	144	51.4%

Chi-square test of independence, chi-square (3) = 15.62, *p* = 0.001, Cramér’s *V* = 0.19.

**Table 3:** Hazard grades of deviant incidents before and after the implementation of legal education.

Grade	Before *n*	Before %	After *n*	After %
Grade 1	653	30.5%	574	50.3%
Grade 2	1,035	48.3%	463	40.5%
Grade 3	453	21.2%	105	9.2%
Total	2,141	100.0%	1,142	100.0%

Chi-square test of independence, chi-square (2) = 150.48, *p* < 0.001, Cramér’s *V* = 0.21.

### 4.6 Ethical considerations

This study was conducted in accordance with the ethical principles for research involving human participants. Informed consent was obtained from all survey respondents before the questionnaire was administered. Respondents were informed that participation was voluntary and that responses would be processed anonymously and used only for aggregated statistical reporting.

## 5 Experiment on the occurrence of deviant events

This section compares the number of deviant incidents, the age distribution of the groups involved, the hazard grades of incidents, and public perceptions of the functions of law between the quarter before and the quarter after the implementation of legal education, evaluating the importance of enhancing public legal awareness in regulating deviant behavior.

### 5.1 The number of cases of deviant incidents

Whether the quality of the people in a city is high, and whether the security of the jurisdiction is good or not can be reflected in the number of deviant incidents in this city. This paper takes province A as an example, and investigates the number of deviant incidents in six randomly selected cities of the province within a quarter. The occurrence of deviant incidents before and after the implementation of legal education is recorded in Figure 4.

**Figure 4:**
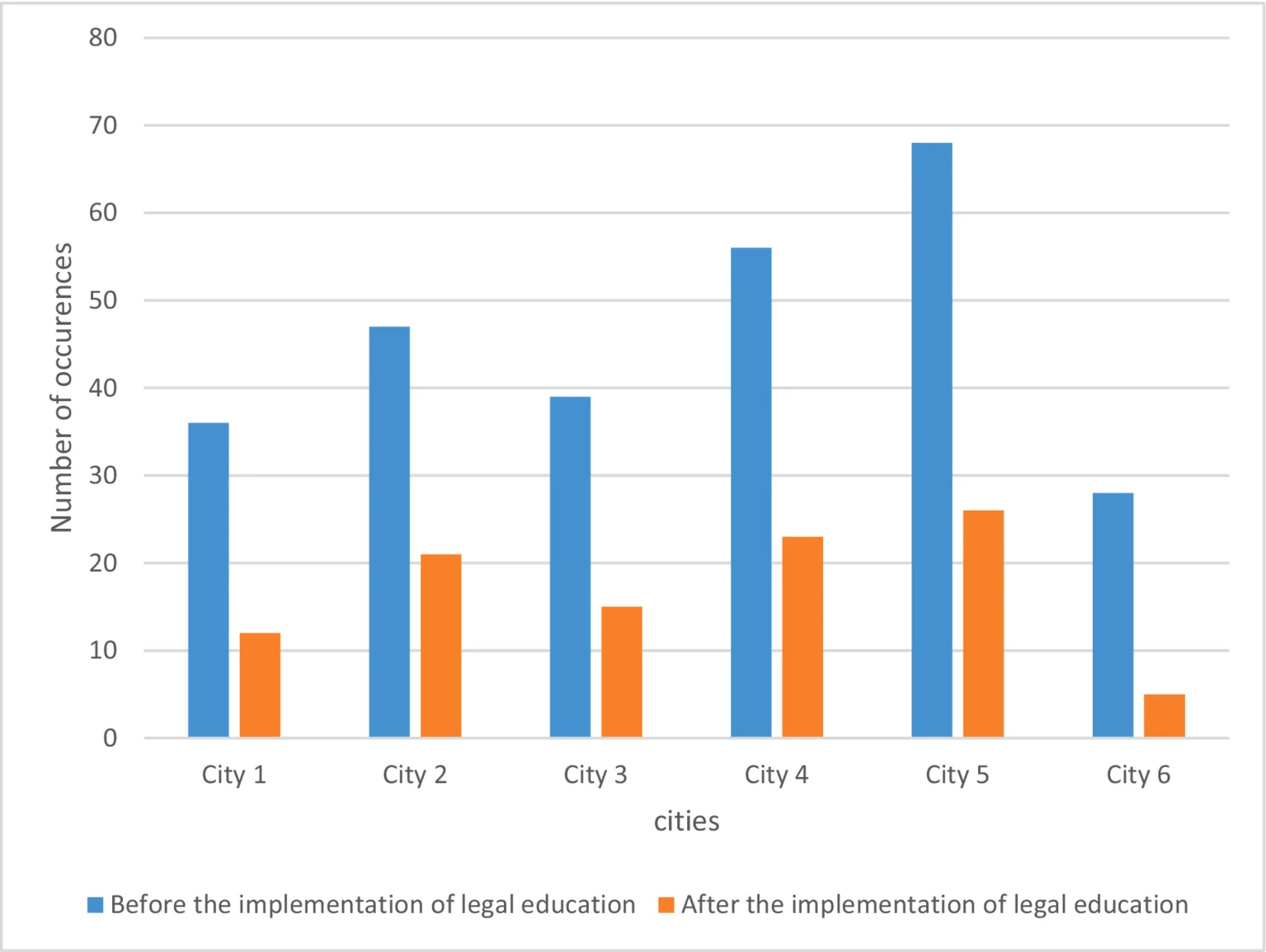
Number of deviant incidents.

Figure 4 and Table 1 present the number of deviant incidents in each of the six cities before and after the implementation of legal education. All six cities showed a reduction in incident numbers, with reduction rates ranging from 55.3 to 82.1%. The paired comparison of the six cities showed a statistically significant reduction, *t*(5) = 9.40, *p* < 0.001, with a 95% confidence interval for the mean reduction of 20.82 to 36.51 incidents and a Cohen’s *d* of 3.84. The Shapiro–Wilk test indicated that the paired differences did not deviate significantly from a normal distribution, *W* = 0.797, *p* = 0.06, supporting the use of the paired-samples *t*-test. The Wilcoxon signed-rank test confirmed the significance of the reduction, *V* = 21, *p* = 0.031, with all six cities showing a decrease in incident numbers. The nonparametric result was consistent with the parametric *t*-test, indicating that the statistical conclusion was robust to the distributional assumption. The total number of deviant incidents across the six cities decreased from 274 in the quarter before the program to 102 in the quarter after the program, a reduction of 62.8%. City 6 showed the largest reduction rate of 82.1%, from 28 incidents to 5 incidents.

The results of these statistical tests are summarized in Table 4.

**Table 4:** Statistical tests for the paired comparison of incident numbers.

Test	Statistic	*p*-value	Interpretation
Paired-samples *t*-test	*t*(5) = 9.40	<0.001	Significant reduction
Shapiro–Wilk (normality of differences)	*W* = 0.797	0.06	Normality not rejected
Wilcoxon signed-rank test	*V* = 21	0.031	Significant reduction (non-parametric)

### 5.2 Groups where deviant incidents occur

Investigating groups where deviant incidents occur can be targeted to control the groups involved, thereby reducing the incidence of deviant incidents. The situation of deviant incident groups in the province before and after the implementation of legal education is recorded in Figure 5.

**Figure 5:**
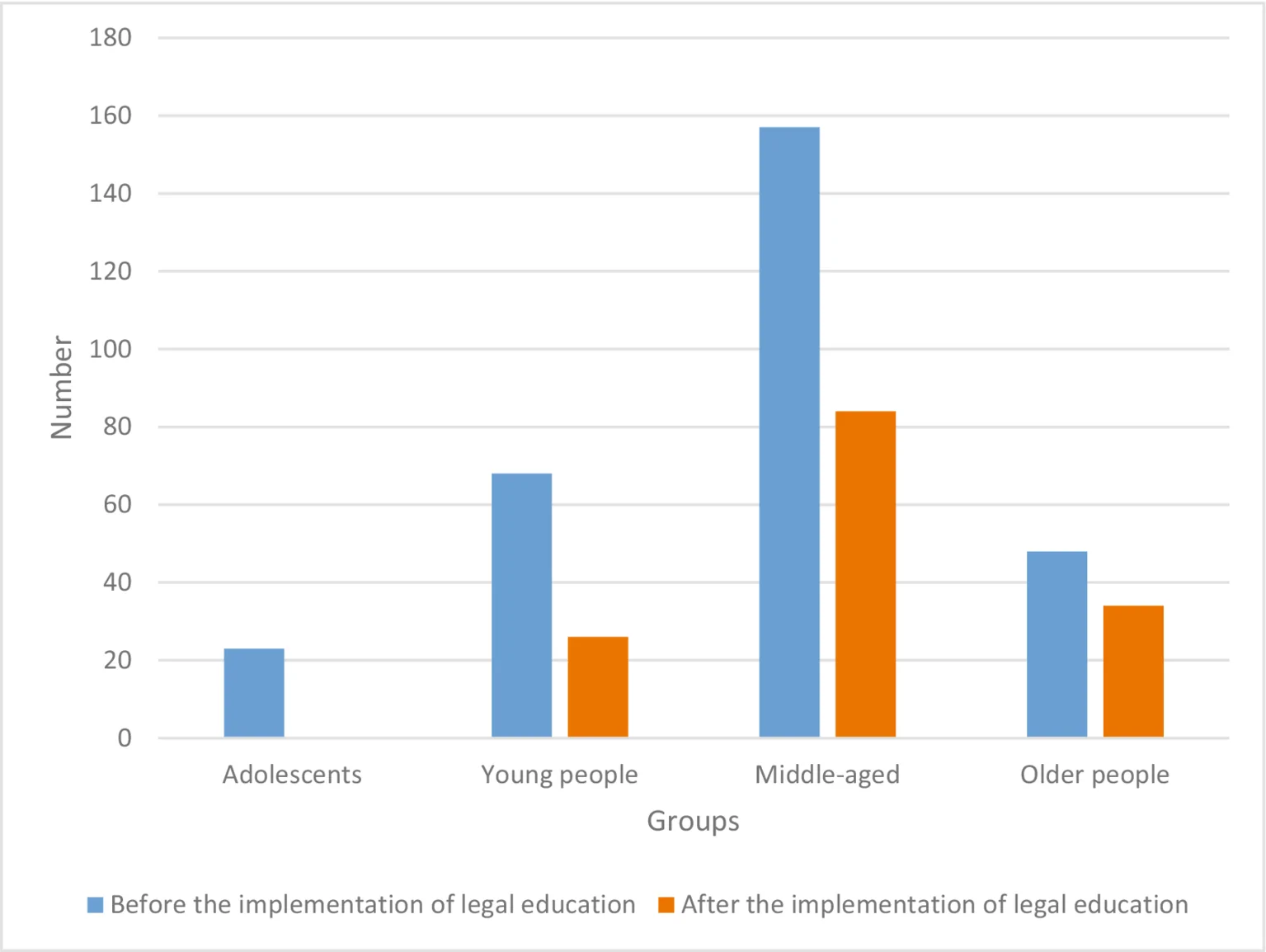
Groups with deviant incidents.

Figure 5 and Table 2 present the age distribution of individuals involved in deviant incidents. Before the implementation of legal education, middle-aged individuals constituted the largest group, followed by young people, older people, and adolescents. The age distribution followed a pattern of higher counts in the middle-age range and lower counts at both ends. After the implementation of legal education, the number of individuals in each age group declined, and the number of adolescents involved in deviant incidents fell to zero. The implementation of legal education is associated with a reduction in deviant incidents across all age groups, and the observed decline provides empirical support for the role of legal education in regulating deviant behavior.

The chi-square test of independence showed a significant association between the implementation of legal education and the age distribution of the groups involved, chi-square (3) = 15.62, *p* = 0.001, Cramér’s *V* = 0.19. The total number of deviant incidents involving all four groups decreased from 296 to 144, a reduction of 51.4%, and the number of incidents involving adolescents fell from 23 to zero.

The group counts describe the number of individuals involved in deviant incidents, aggregated by age group across the six cities. Because a single incident case file may involve multiple individuals, the total number of individuals exceeds the total number of incident case files reported in Table 1. The detailed comparison is presented in Table 2.

### 5.3 The degree of harm of deviant incidents

In social psychology, deviance can be classified into primary deviance and secondary deviance. According to the hazard level, the grades of deviant events can be divided into first, second and third grades. The higher the level, the higher the degree of hazard, and the higher the hazard score. The evaluation criteria for the hazard level of deviant events are recorded in Table 5.

**Table 5:** Grading of deviant incidents.

Grade	Judging criteria	Hazard score
Grade 1	No injuries, ethical issues only	0–60
Grade 2	Not only a moral issue but tends to be criminal	61–90
Grade 3	Criminal behavior	91–100

According to the hazard degree scoring standard in Table 5, the changes in the hazard degree of deviance incidents before and after the implementation of legal education were recorded in Figure 6.

**Figure 6:**
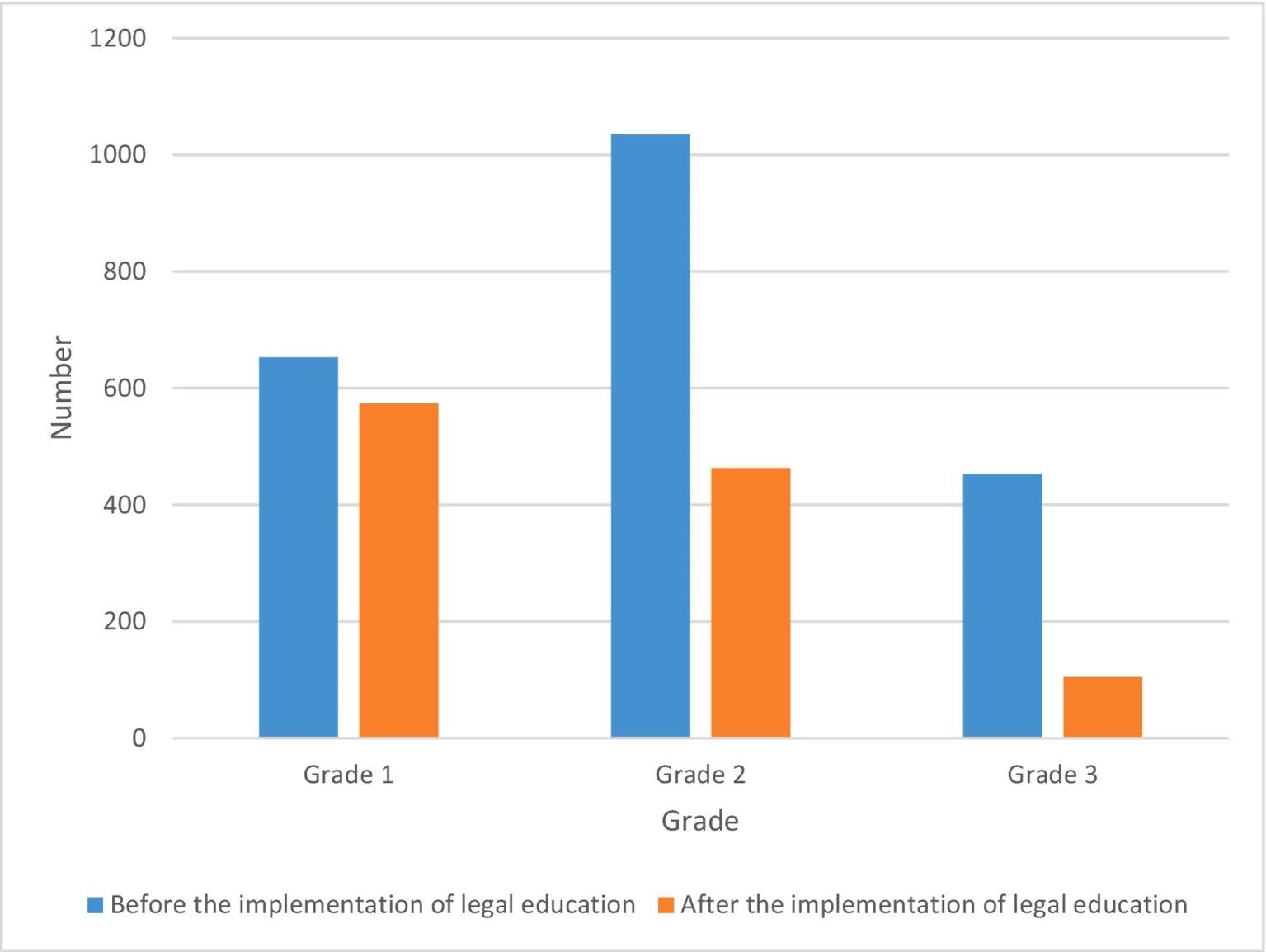
Degree of harm from deviant incidents.

According to Figure 6, the deviant incidents that occurred before the implementation of legal education were mostly at the second-level hazard level, and the degree of harm was higher, while the probability of the first-level and third-level hazard events was lower than that of the second-level hazard events. The concentration of incidents at the second-level hazard grade had a notable adverse impact on society and aroused public dissatisfaction. After the implementation of legal education, the overall number of hazard events decreased, the proportion of first-level hazard events exceeded the combined proportion of second-level and third-level events, and the hazard level of deviant events declined. This pattern indicates that the implementation of legal education is associated with a lower frequency of deviant incidents and a shift toward less severe hazard grades. Before the implementation of legal education, grade-1 incidents accounted for 30.5% of all incidents, grade-2 incidents for 48.3%, and grade-3 incidents for 21.2%; after the implementation of legal education, the corresponding proportions were 50.3, 40.5, and 9.2%. The chi-square test of independence showed a significant association between the implementation of legal education and the distribution of hazard grades, chi-square (2) = 150.48, *p* < 0.001, Cramér’s *V* = 0.21. The hazard-grade statistics record each separate deviant act documented within the incident case files, so the total number of recorded acts exceeds the number of case files reported in Table 1. The total number of incidents decreased from 2,141 to 1,142, a reduction of 46.7%, and the number of grade-1 incidents exceeded the combined number of grade-2 and grade-3 incidents after the program. The detailed comparison is presented in Table 3.

### 5.4 Public opinion on the function of law

There are many functions performed by the law, and different people have different views on the function of the law. In order to understand the true feelings of the public and explore whether the law can be popularized among the public, this article records the public’s views on the legal function before and after the implementation of legal education in Figure 7.

**Figure 7:**
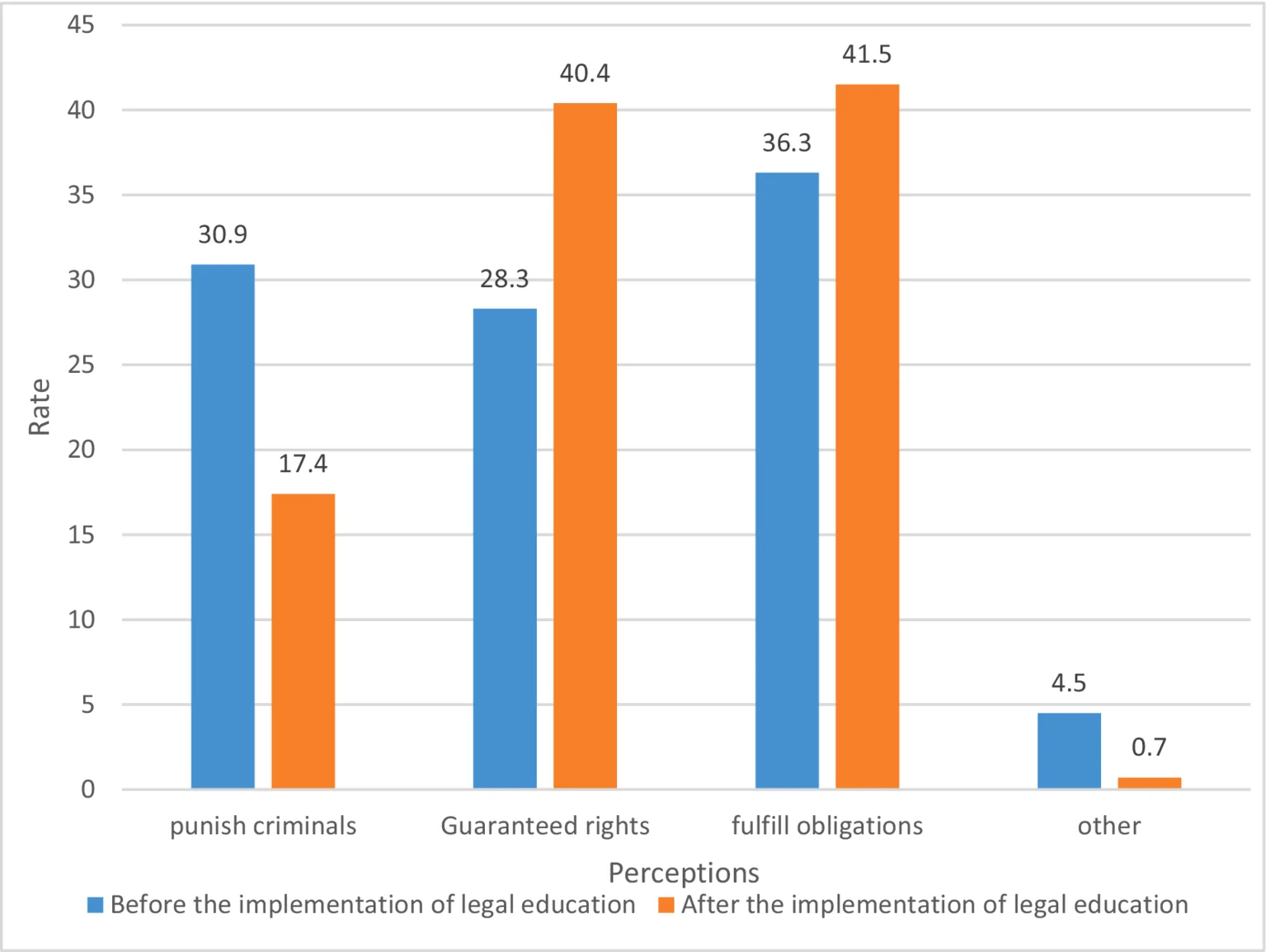
Popular perceptions of legal functions.

As can be seen from Figure 7, before the implementation of legal education, 30.9% of the public believed that the function of the law was to punish criminals, 28.3% of the public believed that the function of the law was to protect rights, 36.3% of the public believed that the function of the law was to perform obligations, and 4.5% of the public held other views. A large number of them believed that the function of the law was to punish criminals. This is a one-sided understanding of the function of the law. The function of the law is not only to punish criminals, but to regulate the behavior of the public and avoid bad events. After the implementation of legal education, the proportion of respondents who perceived the protection of rights as the primary function of law increased from 28.3 to 40.4%, the proportion who perceived the performance of obligations as the primary function increased from 36.3 to 41.5%, and the proportion who perceived the punishment of criminals as the primary function decreased from 30.9 to 17.4%, with the combined proportion of rights protection and obligation performance reaching 81.9%. The shift in public perceptions reflects a change in legal awareness following the implementation of legal education. The shift in public perceptions indicates that legal education is associated with an improvement in legal understanding and legal consciousness.

### 5.5 The effect of legal education

Legal education for the public plays an important role in the improvement of legal awareness and the development of individuals. In order to explore the effect of legal education, this paper analyzes the impact of legal education in this province on the public. Among them, A stands for raising legal awareness, B stands for safeguarding one’s legitimate rights and interests, C stands for consciously fulfilling civic obligations, and D stands for avoiding illegal and criminal acts. The results are recorded in Figure 8.

**Figure 8:**
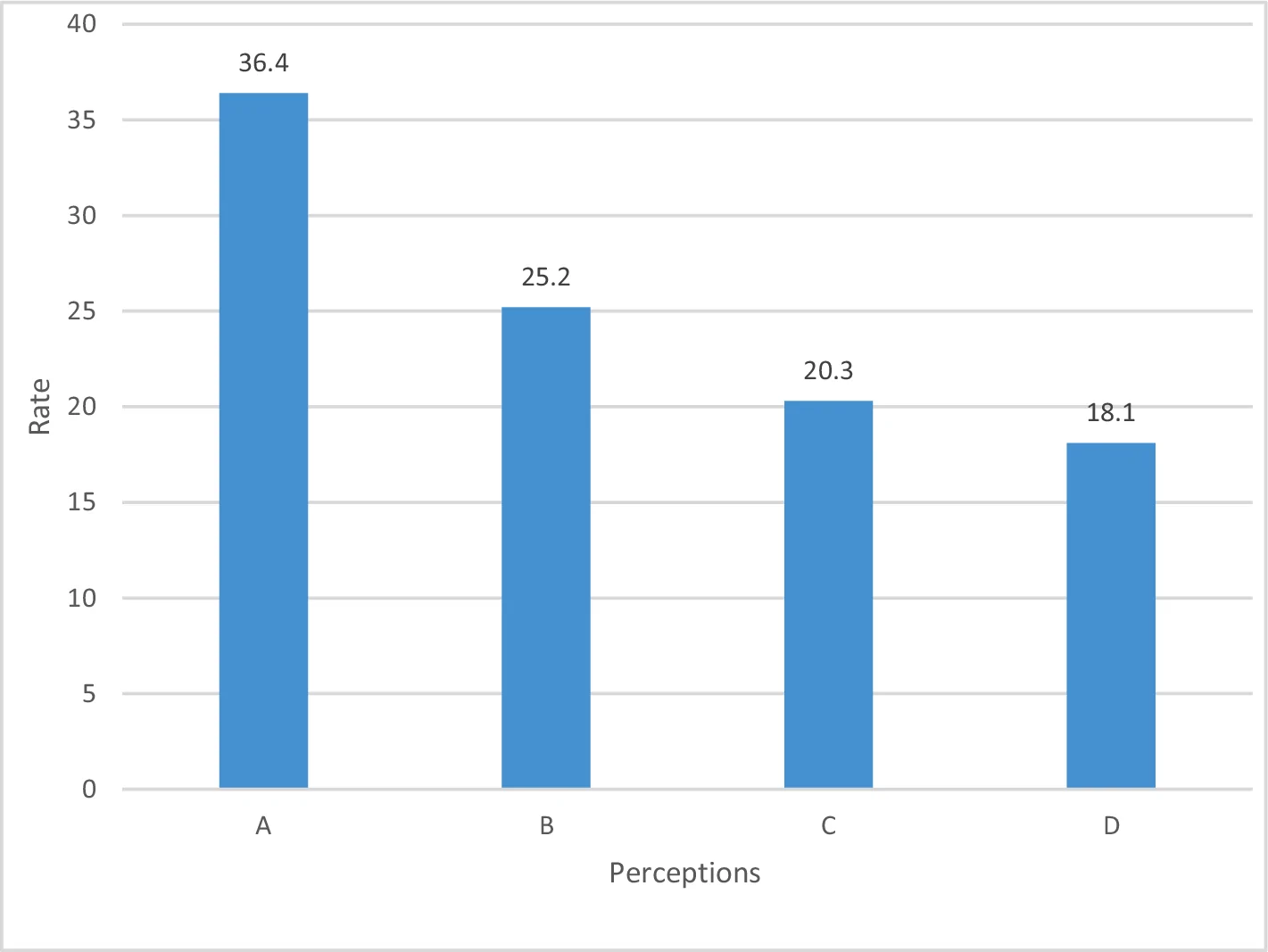
The effect of legal education.

As can be seen from Figure 8, 36.4% of the people believe that legal education has improved legal awareness, 25.2% of the people believe that they safeguard their legitimate rights and interests, 20.3% of the masses believe that they consciously fulfill their civic obligations, and 18.1% of the masses believe that illegal and criminal acts should be avoided. The distribution of perceived effects indicates that legal education is associated with positive changes in legal awareness and lawful conduct.

### 5.6 Psychological constructs and the mechanism of legal education

Table 6 reports the means and standard deviations of the four TPB constructs and self-reported deviant behavior tendency before and after the program.

**Table 6:** The means and standard deviations of the four TPB constructs and self-reported deviant behavior tendency before and after the program.

Construct	Pre *M* (SD)	Post *M* (SD)	*t*(998)	*p*	Cohen’s *d*
Attitude toward legal compliance	4.31 (1.02)	5.22 (0.99)	14.35	< 0.001	0.91
Subjective norm	4.57 (0.95)	5.35 (0.98)	12.76		0.81
Perceived behavioral control	4.66 (0.98)	5.40 (1.03)	11.65		0.74
Behavioral intention	4.64 (1.05)	5.39 (0.97)	11.66		0.74
Deviant behavior tendency	3.71 (0.98)	2.81 (0.99)	−14.42		−0.91

Attitude toward legal compliance increased from 4.31 (SD = 1.02) to 5.22 (SD = 0.99), *t*(998) = 14.35, *p* < 0.001, Cohen’s *d* = 0.91. Subjective norm increased from 4.57 (SD = 0.95) to 5.35 (SD = 0.98), *t*(998) = 12.76, *p* < 0.001, Cohen’s *d* = 0.81. Perceived behavioral control increased from 4.66 (SD = 0.98) to 5.40 (SD = 1.03), *t*(998) = 11.65, *p* < 0.001, Cohen’s *d* = 0.74. Behavioral intention increased from 4.64 (SD = 1.05) to 5.39 (SD = 0.97), *t*(998) = 11.66, *p* < 0.001, Cohen’s *d* = 0.74. Self-reported deviant behavior tendency decreased from 3.71 (SD = 0.98) to 2.81 (SD = 0.99), *t*(998) = −14.42, *p* < 0.001, Cohen’s *d* = −0.91. These results support H1.

Consistent with H2, attitude toward legal compliance (*b* = 0.362, *p* < 0.001), subjective norm (*b* = 0.303, *p* < 0.001), and perceived behavioral control (*b* = 0.251, *p* < 0.001) each positively predicted behavioral intention (*R*^2^ = 0.50).

The mediation analysis supported H3. Legal education (pre/post wave) was significantly associated with attitude toward legal compliance (*b* = 0.912, *p* < 0.001), subjective norm (*b* = 0.778, *p* < 0.001), and perceived behavioral control (*b* = 0.740, *p* < 0.001), and the indirect effect of legal education on self-reported deviant behavior tendency through the TPB constructs was significant (indirect effect = −0.643, 95% bias-corrected bootstrap CI [−0.736, −0.556]). The direct effect of legal education on deviant behavior tendency remained significant (*b* = −0.253, *p* < 0.001), indicating partial mediation. These findings show that the improvement in psychological constructs is a plausible mechanism through which legal education reduces deviant behavior tendency.

## 6 Discussion

The findings demonstrate that the implementation of legal education was followed by substantial reductions in the number of deviant incidents, by a significant change in the age distribution of the groups involved, and by a marked decline in the hazard grades of incidents. The results are consistent with the theoretical account outlined in the introduction, in which legal education transmits legal knowledge and shapes attitudes toward legal norms through the mechanism of legal socialization. The increase in the proportion of respondents who perceived the protection of rights and the performance of obligations as the primary functions of law indicates that legal education altered public cognition of the law, and the reduction of deviant incidents across all age groups is compatible with the mechanism of attitude change specified by the theory of planned behavior.

The findings correspond to previous research on the psychological mechanisms of deviance. Studies of organizational deviance have shown that a sense of entitlement and weakened normative constraints increase transgressive behavior, and the present results suggest that legal education, by strengthening normative constraints, acts in the opposite direction. The observation that the number of deviant incidents involving adolescents fell to zero parallels the finding that high group identification motivates the correction of in-group delinquency, and the improvement of public legal awareness observed here is consistent with research on the psychological mechanisms of prosocial behavior. The decline in hazard grades indicates that the incidents remaining after the program were less harmful, which suggests that legal education reduced not only the frequency of deviant behavior but also its severity.

The mediation findings specify the psychological route of this effect. The improvements in attitude toward legal compliance, subjective norm, and perceived behavioral control jointly translated into a stronger intention to comply with the law, which in turn was associated with a lower tendency toward deviant behavior. This pattern is consistent with the theory of planned behavior, in which attitude, norm, and perceived control shape behavior primarily through behavioral intention, and it extends prior descriptive accounts by identifying the specific constructs that carry the effect of legal education. In particular, the significant role of perceived behavioral control suggests that legal education reduces deviance not only by changing what people think about the law, but also by strengthening their sense of being able to comply with it.

Several limitations of the study merit acknowledgment. The before-after design without a control group does not permit causal attribution, and unmeasured factors that changed between the two quarters constitute an alternative explanation for the observed reductions. The study was conducted in one province, and the transferability of the findings to other legal and cultural contexts requires further examination. The survey measured perceptions through self-report, which is subject to social desirability bias, and the incident statistics relied on official case-filing records, which tend to undercount deviant incidents. Future research with a controlled design and a larger number of cities is needed to strengthen the evidence.

The most significant contribution of this study lies in the demonstration that legal education promotes positive changes in the legal consciousness of the public and fosters more cooperative social relationships, greater respect for legal norms, and a more stable social order. These outcomes support the continued implementation of public legal education programs and the integration of such programs into social governance.

## 7 Importance of enhancing public legal awareness to regulate deviant behavior

### 7.1 Enhancing the public’s legal awareness is the need to change the status quo of the public’s legal awareness

The current state of public legal awareness reflects a gap between legal knowledge and its practical application. Some individuals possess limited legal knowledge and are unable to exercise their legal rights in a procedurally appropriate manner. Others have acquired legal theories yet lack the capacity to apply them in concrete situations, leaving their legitimate rights and interests unprotected when violations occur. Insufficient legal knowledge and weak legal consciousness contribute to engagement in law-breaking behavior, and the diversity of public offenses has increased in recent years.

### 7.2 Enhancing the public’s legal awareness is the need of cultivating people with all-round development

The cultivation of legal awareness constitutes a component of comprehensive personal development, encompassing legal knowledge, legal consciousness, the capacity to apply legal rules, and the formation of legal beliefs. Legal education strengthens the concept of the rule of law, improves legal knowledge, and fosters recognition of legal authority, which in turn promotes voluntary compliance with legal norms and reduces engagement in criminal behavior. When rights and interests are infringed, legally aware individuals are equipped to seek remedies through lawful procedures rather than resorting to extralegal measures, contributing to the maintenance of social order and stability.

### 7.3 Enhancing the public’s legal awareness is the need to create a good social atmosphere

As economic development deepens, the dependence of individuals on legal regulation in production and daily life increases, and the improvement of legal awareness provides a foundation for social stability. Legal publicity and education raise the level of legal knowledge and promote voluntary compliance with laws and regulations. The construction and refinement of the legal system furnish individuals with effective channels for rights protection, and the establishment of institutional rules enhances the capacity to safeguard legitimate interests through lawful procedures. A social environment conducive to legal compliance emerges when individuals adopt law-abiding conduct and contribute to the maintenance of public order.

### 7.4 Enhancing the public’s legal awareness is the need to avoid deviant behavior

Legal provisions define the rights and obligations of citizens, yet insufficient legal education and the persistence of traditional norms lead some individuals to pursue interests through improper means in competitive social contexts. Such behavior harms social order and produces adverse social consequences. Legal education improves legal awareness and is associated with a reduction in deviant behavior, as the findings of the present study indicate.

## 8 Conclusion

This study evaluated the relationship between legal education, public legal awareness, and deviant behavior from the perspective of social psychology, using the theory of planned behavior to identify the psychological mechanism involved. The results show that the implementation of legal education was followed by a significant reduction in the number of deviant incidents, by a significant change in the age distribution of the groups involved, and by a significant decline in the hazard grades of incidents. At the individual level, attitude toward legal compliance, subjective norm, perceived behavioral control, and behavioral intention all improved significantly, and mediation analysis indicated that these psychological constructs mediated the association between legal education and reduced deviant behavior tendency. The findings thus extend the literature by specifying the psychological route—attitude, norm, and perceived control shaping behavioral intention—through which legal education regulates deviant behavior. The before-after design of the study indicates that the observed reductions are associated with legal education rather than conclusively caused by it.

## Data Availability

The original contributions presented in the study are included in the article/supplementary material, further inquiries can be directed to the corresponding author.

## References

[ref1] Ajzen I. (1991). The theory of planned behavior. Organ. Behav. Hum. Decis. Process. 50, 179–211. doi: 10.1016/0749-5978(91)90020-t

[ref2] Ajzen I. (2002). Perceived behavioral control, self-efficacy, locus of control, and the theory of planned behavior 1. J. Appl. Soc. Psychol. 32, 665–683. doi: 10.1111/j.1559-1816.2002.tb00236.x

[ref3] Andrews E. A. (2020). Legal and policy challenges for future marsh preservation in the Chesapeake Bay region. Wetlands 40, 1777–1788. doi: 10.1007/s13157-020-01389-z

[ref4] Bandura A. (1977). Self-efficacy: toward a unifying theory of behavioral change. Psychol. Rev. 84, 191–215. doi: 10.1037/0033-295X.84.2.191, 847061

[ref5] Banerjee D. Gidwani C. Rao T. (2021). The role of "attributions" in social psychology and their relevance in psychosocial health: a narrative review. Indian J. Soc. Psychiatry 36, 277–283. doi: 10.4103/ijsp.ijsp_315_20, 42626699

[ref6] Brannigan A. (2021). The archival turn in classical social psychology: some recent reports. Theory Psychol. 31, 138–146. doi: 10.1177/0959354320920367

[ref7] Cammaerts B. Decillia B. (2020). Journalistic transgressions in the representation of Jeremy Corbyn: from watchdog to attackdog. Journalism 21, 191–208. doi: 10.1177/1464884917734055

[ref9] Fagan J. Tyler T. R. (2005). Legal socialization of children and adolescents. Soc. Justice Res 18, 217–241. doi: 10.1007/s11211-005-6823-3

[ref10] Fang Y. Dong Y. Fang L. (2019). Personality and social psychology honesty-humility and prosocial behavior: the mediating roles of perspective taking and guilt-proneness. Scand. J. Psychol. 60, 386–393. doi: 10.1111/sjop.12551, 31131454

[ref11] Francis J. LaFond R. Olsson P. M. Schipper K. (2004). Costs of equity and earnings attributes. Account. Rev. 79, 967–1010. doi: 10.2308/accr.2004.79.4.967

[ref12] Heckert A. Heckert D. M. (2002). A new typology of deviance: integrating normative and reactivist definitions of deviance. Deviant Behav. 23, 449–479. doi: 10.1080/016396202320265319

[ref13] Humberg S. Nestler S. Back M. D. (2018). Response surface analysis in personality and social psychology: checklist and clarifications for the case of congruence hypotheses. Soc. Psychol. Personal. Sci. 10, 409–419. doi: 10.1177/1948550618757600

[ref14] Ji J. Dimitratos P. Huang Q. (2019). Everyday-life business deviance among Chinese SME owners. J. Bus. Ethics 155, 1179–1194. doi: 10.1007/s10551-017-3542-2

[ref15] Kelty-Stephen D. G. (2017). Threading a multifractal social psychology through within-organism coordination to within-group interactions: a tale of coordination in three acts. Chaos Solitons Fractals 104, 363–370. doi: 10.1016/j.chaos.2017.08.037

[ref16] Kruglanski A. W. Jasko K. Chernikova M. (2017). To the fringe and back: violent extremism and the psychology of deviance. Am. Psychol. 72, 217–230. doi: 10.1037/amp0000091, 28383975

[ref17] Krylova K. O. Jolly P. M. Phillips J. S. (2017). Followers' moral judgments and leaders' integrity-based transgressions: a synthesis of literatures. Leadersh. Q. 28, 195–209. doi: 10.1016/j.leaqua.2016.10.002

[ref18] Liem A. Hall B. J. (2020). Respondent-driven sampling (RDS) method: introduction and its potential use for social psychology research. Jurnal Psikologi Sosial 18, 116–130. doi: 10.7454/jps.2020.13

[ref8] Makwae EN. (2021). Legal frameworks for personnel records management in support of accountability in devolved governments: a case of Garissa County government. Rec. Manag. J. 31, 109–133. doi: 10.1108/RMJ-05-2019-0024

[ref19] Michela C. (2021). Medico-legal aspects of functional neurological disorders: time for an interdisciplinary dialogue. Neurol. Sci. 42, 3053–3055. doi: 10.1007/s10072-021-05162-w, 33811597

[ref20] Paul P. (2021). Existing policies and legal framework for ecotourism in India: a review. Indian For. 147, 936–941. doi: 10.36808/if/2021/v147i10/165716

[ref21] Pitts M. J. (2019). The language and social psychology of savoring: advancing the communication savoring model. J. Lang. Soc. Psychol. 38, 237–259. doi: 10.1177/0261927x18821404

[ref22] Sarita S. (2020). Awareness of intellectual property rights (IPR) among the students of selected colleges affiliated to the Kerala university. Int. J. Informat. Stud. Libraries 5, 61–65.

[ref23] Shuman E. Dan J. Saguy T. (2018). Threat to the group's image can motivate high identifiers to take action against in-group transgressions. Personal. Soc. Psychol. Bull. 44, 1523–1544. doi: 10.1177/0146167218768800, 29756548

[ref24] Singh V. K. (2020). Legal and ethical challenges in multimedia research. IEEE MultiMedia 27, 46–54. doi: 10.1109/mmul.2020.2994823

[ref25] Turel O. (2017). Organizational deviance via social networking site use: the roles of inhibition, stress and sex differences. Pers. Individ. Differ. 119, 311–316. doi: 10.1016/j.paid.2017.08.002, 42574925

[ref26] Tyler T. R. (2003). Procedural justice, legitimacy, and the effective rule of law. Crime Justice 30, 283–357. doi: 10.1086/652233

[ref27] Yam K. C. Klotz A. C. He W. (2017). From good soldiers to psychologically entitled: examining when and why citizenship behavior leads to deviance. Acad. Manag. J. 60, 373–396. doi: 10.5465/amj.2014.0234

